# A Comparison of Two Foldable Phakic Intraocular Lenses Implanted in Different Anatomical Compartments: Artiflex Versus Eyecryl

**DOI:** 10.14744/bej.2021.82612

**Published:** 2021-09-27

**Authors:** Bulent Kose, Alper Agca

**Affiliations:** 1. Department of Ophthalmology, Aritmi Osmangazi Hospital, Bursa, Turkey; 2. Department of Ophthalmology, Dünya Göz Hospital, Istanbul, Turkey

**Keywords:** Myopia, phakic intraocular lenses, refractive surgery

## Abstract

**Objectives:**

The aim of this study was to compare the refractive results and safety of Artiflex (Ophtec BV, Groningen, Netherlands) and Eyecryl (BioTech Healthcare GmbH, Luzern, Switzerland) phakic intraocular lenses (pIOL).

**Methods:**

The medical records of patients who underwent implantation of Artiflex or Eyecryl pIOL were retrospectively reviewed. Patients with a follow-up of 3 years were included in the study. Manifest refractive error, uncorrected and corrected visual acuity, intraocular pressure (IOP) and central endothelial cell density (ECD) data were evaluated preoperatively and at 1 and 3 years after surgery.

**Results:**

In all 79 eyes (Artiflex group: 35 eyes; Eyecryl group: 44 eyes) were included in the study. The preoperative spherical equivalent (SE) of manifest refractive error was -11.53±3.46 in the Artiflex group and -13.08±3.01 in the Eyecryl group. Three years after the operation, the efficacy index was 1.06±0.55 and 1.15±0.85 in the Artiflex and Eyecryl groups, respectively. The safety index was in 1.32±0.49 and 1.46±0.95 the Artiflex and Eyecryl groups, respectively. The SE refractive error, efficacy index, safety index UDVA, CDVA, IOP, and ECD were not significantly different between groups during follow-up.

**Conclusion:**

Both the Artiflex and Eyecryl foldable pIOLs were found to be safe and effective up to 3 years after implantation. Prospective longitudinal studies are needed to assess and compare the rate of cataract formation.

## Introduction

Implantation of a phakic intraocular lens (pIOL) is an alternative to corneal refractive surgical procedures such as photorefractive keratectomy, laser in situ keratomileusis, and small incision lenticule extraction (1-6). It is usually preferred in high myopia because the quality of vision decreases, and the complication rate increases after a certain degree with corneal refractive surgical procedures. There are three types of pIOLs: Angle supported, iris-claw, and posterior chamber (2).

Several designs of angle-supported IOLs have been abandoned because of high rates of complications such as cataracts, glaucoma, and excessive endothelial cell loss in the long term. In contrast to the angle-supported IOLs, two iris-claw pIOLs Artisan (Ophtec BV, Groningen, the Netherlands) and Artiflex (Ophtec BV, Groningen, the Netherlands) have a long history and are considered to have good safety and efficacy (5). Although they are anterior chamber pIOLs, they differ from the angle-supported pIOLs is that they have no contact with any intraocular structure other than the iris. The optic of the IOL is polysiloxane and the haptics are PMMA.

Posterior chamber pIOLs are designed to be placed in the posterior chamber behind the iris with the haptic zone resting on the ciliary sulcus. One posterior chamber pIOL has a long history of follow-up and met the efficacy and safety criteria of the US Food and Drug Administration (FDA) (6). However, Eyecryl pIOLs are a new posterior chamber pIOL and its long-term results are not established in the literature.

The purpose of this study was to compare clinical results and post-operative complications two pIOLs, both of which have the advantage of being foldable but made up of different materials and implanted in different anatomical locations with different surgical techniques.

## Methods

The study adhered to the tenets of the Declaration of Helsinki and approval was obtained from the Okmeydani Training and Research Hospital review board (February 5, 2020, No: 13). The medical records of patients who received Eyecryl or Artiflex pIOL implantation in our clinic were retrospectively reviewed and patients with a follow-up of at least 3 years were included in the study. Preoperatively, all patients received uncorrected distance visual acuity (UDVA) and corrected distance visual acuity (CDVA) distance visual acuity measurement, slit-lamp examination, intraocular pressure measurement (Goldman applanation tonometry), fundoscopy, corneal topography examination (Sirius Corneal Topography and Aberrometry System, Costruzioni Strumenti Oftalmici, Italy), and central endothelial cell density (ECD) measurement using a specular microscope (CEM 530, NIDEK, Japan). The patients were scheduled for yearly follow-up after the 1st year of surgery. All pre-operative examinations were repeated in yearly follow-up visits, which is routine in our clinic. A lens opacity that results in the loss of ≥2 lines of CDVA during follow-up was defined as cataract.

### Artiflex pIOLs Implantation

After sterile surgical draping and subtenon anesthesia, two paracenteses of 1.2 mm were performed on two sides of the planned (superior) main incision. 0.01% acetylcholine (Miochol-EO, Novartis) was injected into the anterior chamber. The anterior chamber was filled with an ophthalmic viscosurgical device (OVD) (Provisc, Alcon) and a 3.2 mm main incision centered at 12 o’clock is performed with a slit knife. The Artiflex pIOL was introduced from the main incision into the anterior chamber using the special implantation tool provided by the manufacturer and it was rotated inside the eye until it is horizontal. One IOL haptic was grasped with specially designed forceps introduced from the main incision, and the iris beneath the haptic was enclaved in the claws of the pIOL using a special needle introduced from the paracentesis on that side. The main incision and the opposite paracentesis were used in a similar manner for enclavation of the iris on the other side. Iridotomy was performed, and the incisions were hydrated with balanced salt solution (BSS).

### Eyecryl pIOLs Implantation

The pupil was dilated with cyclopentolate, and phenylephrine drops, instilled 30 min prior to surgery. After subtenon anesthesia, two paracentesis incisions were performed on two sides of the planned (temporal) main incision and adrenalin was injected into the anterior chamber. Then, the IOL was loaded into the cartridge/injector system and a cohesive OVD (Provisc, Alcon) was injected into the anterior chamber. A 2.8 mm clear corneal tunnel incision was performed and the Eyecryl pIOL was introduced into the anterior chamber (over the iris) through the incision in a horizontal position. The IOL was gently positioned in the sulcus, using a push-pull introduced from a paracentesis incision. The remaining OVD was completely washed out of the anterior chamber with a BSS and the incisions were hydrated with BSS. No pre-operative or intraoperative peripheral iridectomies were performed.

### Statistical Methods

Statistical analysis was performed using SPSS for Windows (version 21.0; IBM, Armonk, NY), and the associated graphics were generated with Microsoft Excel 2013 (Microsoft Corporation, Seattle, WA, USA). The mean and standard deviation were used to report variables. The variable distribution was determined using the Shapiro–Wilk test. Repeated measures analysis of variance was used to evaluate variables during follow-up when more than 2 visits were compared. A paired t-test was used to compare two variables at two different visits. Categorical variables were compared with Chi-square and Fisher’s exact tests. P<0.05 was considered statistically significant.

## Results

Seventy-nine eyes of 44 patients were included in the study. There were 44 eyes in Eyecryl Group and 35 eyes in Artiflex Group. In all patients, pre-operative central ACD was ≥3.00 mm (from endothelium). Visual acuity, refraction, and ECD at pre-operative visit and post-operative 1 and 3 years are listed in Table 1. Although manifest refraction spherical equivalent (SE), UDVA, CDVA, and ECD were not significantly different between the groups at any visit, mean astigmatism was significantly higher in Artiflex group, preoperatively. At post-operative 1 and 3 years, UDVA, CDVA, manifest refraction SE, cylinder, or ECD were not statistically significant between the groups (Table 1).

**Table 1.** Outcome parameters over the course of the study

**Manifest Refraction SE (D) (Mean±SD)**	**Preoperative**	**1 year**	p*	**3 years**	p**
Artiflex	-11.53±3.46	-0.54±0.78	<0.001	-1.23±1.08	<0.001
Eyecryl	-13.08±3.01	-0.42±0.59	<0.001	-0.93±0.87	<0.001
P***	0.037	0.444		0.180	
**Astigmatism (D) (Mean±SD)**	Preoperative	1 year	p*	3 years	p*
Artiflex	1,49±1.00	1.33±0.84	0.214	1.21±0.84	0.147
Eyecryl	1.00±0.67	0.89±0.73	0.271	1.45±0.78	<0.001
P**	0.018	0.016		0.196	
**Corrected Distance visual acuity (logMAR) (Mean±SD)**	**Preoperative**	**1 year**	p*	**3 years**	p*
**Artiflex**	**0.26±0.16**	**0.19±0.12**	**0.003**	**0.17±0.11**	**0.272**
Eyecryl	0.29±0.18	0.19±0.21	0.002	0.17±0.18	0.216
P**	0.573	0.951		0.899	
**Central ECD (Cells/mm^2^) [Mean±SD (% of cumulative ECD loss)]**	**Preoperative**	**1 year**	p*	**3 years**	p*
Artiflex	2681±275 (N/A)	2599±242 (3.05)	0.930	2534±238 (5.48)	<0.001
Eyecryl	2656±270 (N/A)	2575±253 (3.04)	0.04	2512±251 (5.42)	0.260
P**	0.692	0.670		0.678	

*: Paired samples T-Test compared to previous visit; **: Independent samples T-Test.

**Figure 1.**
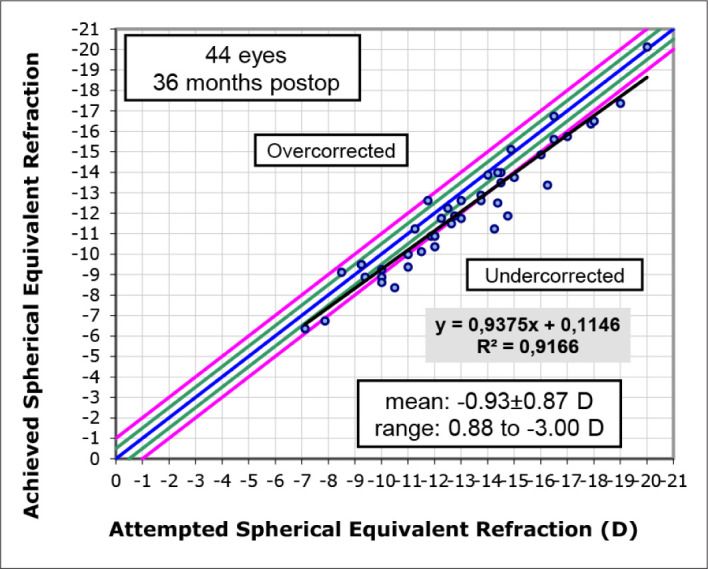
Attempted versus achieved spherical equivalent refraction in Eyecryl group.

**Figure 2.**
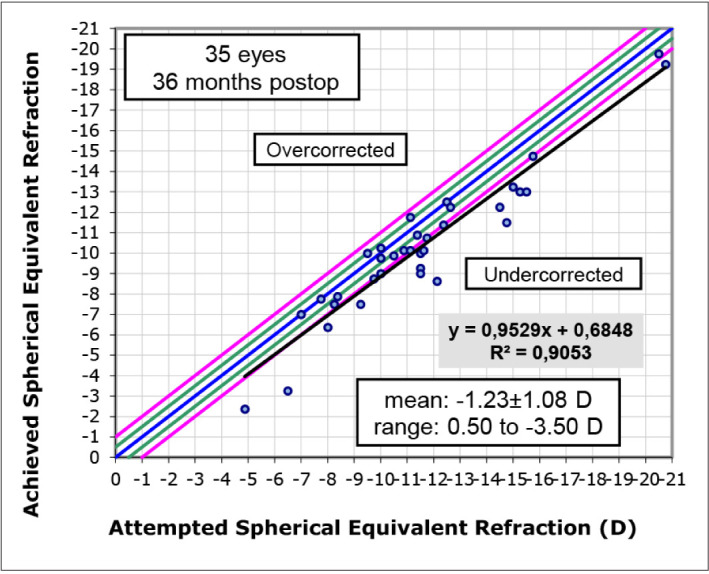
Attempted versus achieved spherical equivalent refraction in Artiflex group.

**Figure 3.**
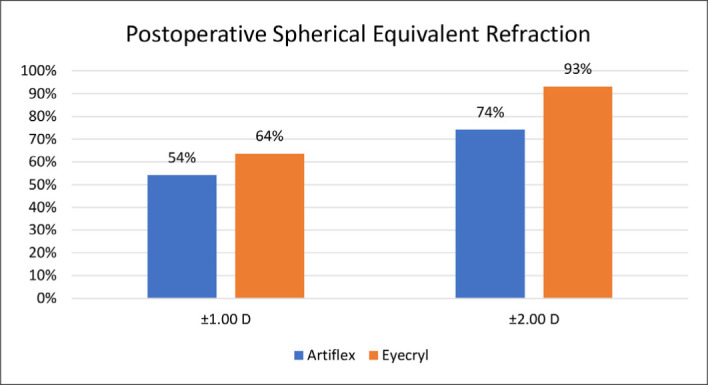
Spherical equivalent of mean manifest refraction.

Figures 1 and 2 show a scatterplot of attempted versus achieved manifest refraction SE at 3-year visit in Eyecryl and Artiflex groups, respectively. At post-operative 3 years, 64% and 54% of patients were within ±1.00 D of emmetropia in in Eyecryl and Artiflex groups, respectively [(Fig. 3), Pearson Chi-square, p=0.491]. However, significantly higher number of patients were in within ±2.00 D of emmetropia Eyecryl group at post-operative 3 years [(Fig. 3), Pearson Chi-square, p=0.028].

Efficacy index (Pre-operative CDVA/Post-operative UDVA) was not significantly different between the groups at 3 years (1.15±0.85 in Eyecryl and 1.06±0.55 in Artiflex groups; t-test, two tailed p=0.586). Safety index (Pre-operative CDVA/Post-operative UDVA) was 1.46±0.95 and 1.32±0.49 in Eyecryl and Artiflex groups, respectively, t-test, two tailed p=0.429. Anterior subcapsular cataract developed in two eyes of one patient in Eyecryl group. Anterior subcapsular cataract developed in two eyes of a patient in Eyecryl group. This patient lost two lines of CDVA in both eyes. However, this patient underwent cataract surgery in only one eye because he was satisfied with his vision was after cataract operation. The opacity and the visual acuity were stable in the fellow eye at the last follow-up. One eye in Artiflex group developed cataract, however, loss of CDVA was only one line and cataract surgery was not performed. Rhegmatogenous retinal detachment occurred in one eye of a patient in Eyecryl group. None of the eyes developed glaucoma. No other serious complications with the potential to affect CDVA were observed in both groups.

## Discussion

A limited number of studies have previously compared pIOLs implanted in different anatomical compartments (1-4). However, all these studies evaluated Artiflex and implantable Collamer lens. A comparison of implanted Collamer lens or Artiflex with Eyecryl has not been performed before.

There was a statistically significant difference between the mean pre-operative astigmatism values between the groups. However, the difference was clinically small (0.50 D), and therefore, we believe that its effect on visual acuity is negligible. Furthermore, the mean pre-operative and post-operative SE, UDVA, and CDVA were similar between the groups. The mean post-operative astigmatism was not significantly different at the last post-operative visit. This was due to an approximately 0.50 D increase in with the rule astigmatism in Eyecryl group. The increase is probably due to the standard temporal 2.75 mm incision in this group. A temporal incision was performed in Eyecryl groups as implantation is easier with this approach and it was the manufacturers advice. However, changing the position of the incision depending on the astigmatism depending on the subjective or corneal astigmatism may be a better option.

We found that mean SE was similar (approximately −0.5 D) in both groups during follow-up. However, when 1-year and 3-year results are compared, there was a statistically significant regression in the refractive effect in both groups (Table 1). Regression of the refractive effect is not surprising after implantation of a pIOL because most of these patients have axial progressive myopia. The increase in SE during follow-up is probably due to an increase in axial length (AL). However, this was a retrospective study and AL measurement was not a part of our routine postoperative examinations. Thus, we could not perform an analysis to study the correlation between the change in SE and the change in AL. Other studies in the literature also report that the post-operative SE increases during follow-up (5,6).

Despite only 64% and 54% of patients were within ±1.00 D of emmetropia in in Eyecryl and Artiflex groups, we found that efficacy indices were above 1.00 for both pIOLs and the difference between them was not statistically significant. In other words, mean post-operative uncorrected visual acuity was better than mean pre-operative corrected visual acuity for both pIOLs. The high efficacy index despite residual refractive errors probably results from the significantly improved CDVA after surgery in both groups. An increase in CDVA after correction of a high refractive error with pIOL implantation is a well-known phenomenon (7-9). Moreover, our results are in line with previous reports (9-11).

One eye in Eyecryl group developed a rhegmatogenous retinal detachment at post-operative 2^nd^ year. Rhegmatogenous retinal detachment after pIOL implantation has been reported before (12-14). Bamashmus et al.(14) reported an RD rate of 0.32% in 617 eyes. However, it is unknown whether pIOL implantation in patients with high myopia induces iatrogenic vitreous changes that could increase the incidence for RD. However, Kohnen et al.(12) considered RDs after pIOL implantation as a part of the natural history of the RD in cases of high myopia because the incidence of retinal detachment is higher in highly myopic eyes when compared to emmetropic eyes. However, intraocular surgery can further increase this risk by inducing PVD or other mechanisms (7,15,16).

Cataract formation is a potential complication of iris-claw and posterior chamber pIOLs. Guber et al.(17) reported an incidence of cataract after implantable contact lens (ICL) implantation of 4.9% after 5 years and 18.3% after 10 years. During our follow-up of 3 years, cataracts developed in 2 eyes (4.5%) of a patient in Eyecryl group which has a similar design to ICL. Although these rates seem similar, a direct comparison between posterior chamber pIOLs of different designs does not yet exist in the literature and the true incidence of cataract formation after Eyecryl pIOL implantation can be revealed only after a prospective study with a control group. None of the eyes in Artiflex group developed cataracts in our study, however, it is known that iris-claw implantation also increases the rate of cataract formation (18). Jonker et al.(18) reported that the explantation rate of iris-fixated pIOLs was 12% after almost 14 years of follow-up, with 59% of pIOL explantation caused by cataract formation and 32% caused by endothelial cell loss. In another study, 10% of underwent cataract surgery during 10 years follow-up (19). However, cataract formation rate may be higher for posterior chamber pIOLs when compared to iris-claw lenses.

Endothelial cell densities were similar in both groups preoperatively and postoperatively. Cumulative endothelial cell loss at 3 years was 5.48% and 5.42% in Artiflex and Eyecryl groups, respectively. These results indicate a decrease in ECD in the 1st year and a stabilization thereafter, however, 3 years are a short follow time to draw conclusions about rate of endothelial cell loss. There are no long-term studies reporting endothelial cell loss after Eyecryl pIOL implantation. Eyecryl lens has a very similar design and implanted in the same anatomical location with ICL. Edelhauser et al.(20) performed non-contact specular microscopy to evaluate the 3–4 years effects of the ICL on the corneal endothelium as a subgroup study in a Phase III U.S. FDA clinical trial. They reported that the cell loss between 1 year and 3 years is most readily explained by prolonged corneal remodeling following the surgical procedure rather than ongoing cell loss. To the best of our knowledge, there is no reported case of endothelial decompensation after ICL implantation, except for a case that developed endothelial decompensation after trauma and dislocation (21). However, late endothelial decompensation after implantation of iris-claw IOLs (Artisan and Artiflex) is reported, which indicate progressive cell loss in at least some patients (22,23). Thus, posterior chamber pIOLs may be safer for endothelium.

The main disadvantage of this study is its retrospective nature. A correlation of AL changes and regression was not possible due to this. Furthermore, due to the routine workflow of our clinic, post-operative examinations are performed by different residents or ophthalmologists at different times. Thus, a new but mild lens opacity which does not result in a decrease in visual acuity, a mild pigment dispersion or cells in the early post-operative period which does not have a clinical significance may have gone unnoticed or may have not been reported in patient files in both groups. This weakness of the study should be considered when interpreting the results.

## Conclusion

In conclusion, we compared two foldable pIOLs made up of different materials and implanted in different anatomical compartments. We found both highly effective at 3-year follow-up. Visual acuities and endothelial cell densities were similar between the groups. Although cataract surgery was performed in one eye in Eyecryl group, the rate of cataract formation was similar to other posterior chamber pIOLs in the market. A prospective comparative study with a control group is needed to reveal relative rates of cataract formation after implantation of different pIOLs.

## Disclosures

### Ethics Committee Approval:

Okmeydanı Training and Research Hospital. Date: 07/01/2020, No:13.

### Peer-review:

Externally peer-reviewed.

### Conflict of Interest:

None declared.

### Authorship Contributions:

Involved in design and conduct of the study (AA, BK); preparation and review of the study (AA, BK); data collection (AA, BK); and statistical analysis (AA, BK).
